# ESTABLISHING THE VALIDITY OF SIMULATION TO CAPTURE ELDERSPEAK COMMUNICATION IN DEMENTIA CARE

**DOI:** 10.1093/geroni/igac059.530

**Published:** 2022-12-20

**Authors:** Clarissa Shaw, Katie Knox, Heather Bair, Erica Watkinson, Delaney Weeks, Paige Hanson

**Affiliations:** University of Iowa, Iowa City, Iowa, United States; University of Iowa College of Nursing, Iowa City, Iowa, United States; University of Iowa College of Nursing, Iowa City, Iowa, United States; University of Iowa College of Nursing, Iowa City, Iowa, United States; University of Iowa College of Nursing, Iowa City, Iowa, United States; University of Iowa College of Nursing, Iowa City, Iowa, United States

## Abstract

Using simulation may be an innovative approach for evaluating dementia care in research and education. The purpose of this study was to determine if communication in the simulated environment is congruent to communication in the naturalistic environment. Nursing staff (n=10) were each audio-recorded caring for an actual hospitalized person living with dementia (PLWD), and in three simulations evaluating the use of a manikin, young nursing assistant, and trained older adult standardized patient acting as the PLWD. The audio-recordings were coded for elderspeak (i.e., infantilizing) communication. Reactions to each simulation were gathered and qualitatively analyzed. The amount of elderspeak and neutral communication in the actual care encounters did not significantly differ (p>.05) from the communication in any of the three simulations indicating that simulation may be a valid method to capture elderspeak by nursing staff. The nursing staff indicated that the standardized patient provided the most realistic simulation.

